# Primary solitary fibrous tumor of the liver: an uncommon neoplasm

**DOI:** 10.1093/gastro/goaf012

**Published:** 2025-02-20

**Authors:** Shu Sasaki, Luis Veloza, Christine Sempoux, Didier Sarazin, Emilie Uldry, Nermin Halkic, Ismail Labgaa

**Affiliations:** Department of Visceral Surgery, Lausanne University Hospital (CHUV), Lausanne, Switzerland; Faculty of Biology & Medicine (FBM), University of Lausanne (UNIL), Lausanne, Switzerland; Faculty of Biology & Medicine (FBM), University of Lausanne (UNIL), Lausanne, Switzerland; Department of Pathology, Lausanne University Hospital (CHUV), Lausanne, Switzerland; Faculty of Biology & Medicine (FBM), University of Lausanne (UNIL), Lausanne, Switzerland; Department of Pathology, Lausanne University Hospital (CHUV), Lausanne, Switzerland; Department of Pathology, Viollier Weintraub SA, Geneva, Switzerland; Department of Visceral Surgery, Lausanne University Hospital (CHUV), Lausanne, Switzerland; Faculty of Biology & Medicine (FBM), University of Lausanne (UNIL), Lausanne, Switzerland; Department of Visceral Surgery, Lausanne University Hospital (CHUV), Lausanne, Switzerland; Faculty of Biology & Medicine (FBM), University of Lausanne (UNIL), Lausanne, Switzerland; Department of Visceral Surgery, Lausanne University Hospital (CHUV), Lausanne, Switzerland; Faculty of Biology & Medicine (FBM), University of Lausanne (UNIL), Lausanne, Switzerland

## Introduction

Solitary fibrous tumors (SFTs) are rare mesenchymal neoplasms that are characterized by spindle cell proliferation in association with a network of thin branching vessels (staghorn) [1]. They commonly present as slow-growing masses and can reach large sizes before becoming symptomatic. SFTs usually present in middle age, peak in the fifth and sixth decades, and are rare in children. Prevalence is equal in men and women [2]. SFTs can occur in any organ, but primary SFTs of the liver are particularly rare [3]. We presented the case of an 86-year-old woman who was diagnosed with a primary solitary fibrous tumor of the liver that was successfully resected by using laparoscopic left lateral segmentectomy. This report focuses on primary SFTs of the liver and reviews the relevant literature.

## Case report

**Figure 1.**
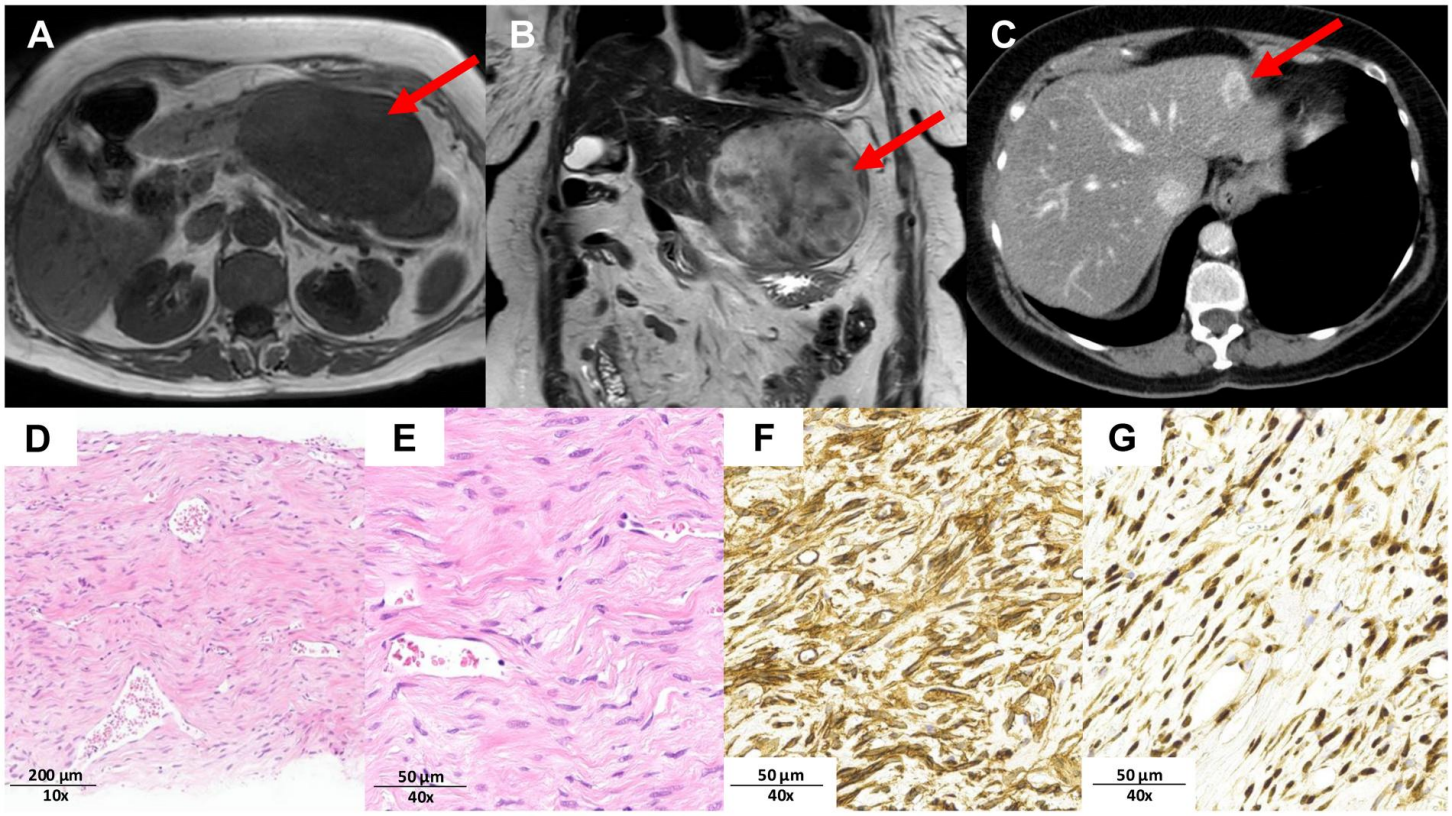
Preoperative imaging examinations and pathological findings of the tumor. (A) Preoperative abdominal magnetic resonance imaging (MRI) shows a 12 cm × 7 cm liver mass of the left lateral segment, hypointense on T1-weighted imaging. (B) Heterogeneously enhanced on T2-weighted imaging. (C) Abdominal CT showing the mass measuring 2.5 cm, 11 years ago. (D) Hematoxylin and eosin staining of the liver biopsy showing a spindle cell tumor proliferation arranged around branching and dilated vasculature within collagenous stroma (×10). (E) Hematoxylin and eosin staining showing a spindle cell tumor proliferation arranged around branching and dilated vasculature within collagenous stroma (×40). (F) Tumor cells are diffusely positive for CD34 (×40). (G) They show strong and diffuse nuclear staining for STAT6 (×40).

An 86-year-old woman presented with pain of the left upper quadrant (LUQ). She had a history of bilateral breast cancer treated with surgery and adjuvant chemo–radio–hormono-therapy 15 years previously. Physical examination showed a firm mass of the LUQ, with no tenderness rebound. Laboratory tests were unremarkable. Abdominal magnetic resonance imaging (MRI) revealed a 12 cm × 7 cm liver mass of the left lateral segment, hypointense on T1-weighted imaging (Figure 1A) and heterogeneously enhanced on T2-weighted imaging (Figure 1B). Retrospectively, the mass had already been visible on a computed tomography (CT) scan 11 years previously, measuring 2.5 cm (Figure 1C). As imaging features are not definitive, metastatic liver tumor was one of the differential diagnoses, considering the past medical history of breast cancer. A percutaneous biopsy of the tumor was obtained. Histopathological analysis showed a spindle cell tumor proliferation disposed in a dense collagenous stroma, admixed with branching and hyalinized blood vessels (Figure 1D and E). No mitosis or necrosis was observed. On immunohistochemistry, the tumor cells showed strong and diffuse positivity for CD34 (Figure 1F) and nuclear STAT6 (Figure 1G), whereas EMA, S100, HMB-5, SMA, desmin, CD31, ERG, and CD117 were negative. The histological findings together with the immunohistochemical profile (CD34+, STAT6+) were consistent with the diagnosis of SFT of the liver. The patient underwent laparoscopic left lateral segmentectomy with an uneventful post-operative course. Pathological analysis of the surgical specimen revealed the presence of a well-circumscribed and partially encapsulated mass of 12 cm, surrounded by normal liver. The histological analysis of the specimen also confirmed the diagnosis of SFT. The patient will be followed up with imaging examinations.

## Discussion

Primary SFTs of the liver is particularly rare, thus clinical features and behaviors are not well described. SFTs of the liver typically appear as low-density masses with heterogeneous post contrast enhancement that persists on delayed phases on CT [4] and heterogeneously enhancing with a more readily apparent capsule on MRI [5]. The most common differential diagnoses are cholangiocarcinoma, fibrolamellar hepatocellular carcinoma, hepatic sclerosing and sclerosed hemangioma, or spindle cell/fibrous mesothelioma. Imaging features of SFT are not definitive and the diagnosis is thus challenging. It essentially relies on histological and immunohistochemistry analysis on the preoperative biopsy or the resected specimen. The expression of CD34 is strong and diffuse in >80% of SFTs [6]. But signal transducer and activator of transcription 6 (STAT6) immunohistochemistry positive staining is a surrogate of NAB2-STAT6 gene fusion, which is highly sensitive and specific for this entity [7]. Surgical resection is the treatment of choice for localized or oligometastatic SFTs [8]. Although cases of recurrence or metastasis have been reported, most SFTs of the liver (>80%) are benign and cured by surgical resection [3]. Advanced age, larger tumor size, infiltrative margins, high cellularity, prominent cellular atypia, tumor necrosis, increased mitotic activity, and Ki-67+ cell density have been proposed as prognostic factors for localized resected SFT cases [8, 9]. According to the risk prediction model of Demicco *et al.* that uses four factors (age, tumor size, mitotic count, and tumor necrosis) [10], the present SFT was classified as being of low risk (score 3 out of 7). But risk factors that aer specific to the primary SFTs of the liver are not proposed due to little experience. Primary SFT of the liver is a rare neoplasm that should be considered in the differential diagnosis for slowly growing large hepatic tumors. (Informed consent was obtained from the patient to publish these images.)

## Authors’ Contributions

Study concept and design: S.S., I.L. Acquisition of data: S.S., L.V., D.S., C.S., I.L. Analysis and interpretation of data: S.S., L.V., C.S., D.S., E.U., N.H., I.L. Drafting of the manuscript: S.S., L.V., C.S., I.L. Critical revision of the manuscript for important intellectual content: S.S., L.V., C.S., D.S., E.U., N.H., I.L.
